# A Wolf in Sheep's Clothing: A Case of Dilated Cardiomyopathy Presenting with Nonspecific Digestive Symptoms: Insights into Nonocclusive Mesenteric Ischemia

**DOI:** 10.1155/2011/406832

**Published:** 2011-10-25

**Authors:** Antoine Kossaify

**Affiliations:** Cardiology Division, NDS-USEK University Hospital, St. Charbel Street, Byblos, Lebanon

## Abstract

We report on a 32-year-old male patient who presented to the emergency room for abdominal pain associated with nausea and vomiting. The patient experienced these symptoms for the last 3 months and was taken in charge on an outpatient basis. Assessment in the emergency room showed hemodynamic collapse, there were no signs of acute surgical abdomen. Emergent cardiac echogram showed severely dilated hypokinetic cardiomyopathy. The diagnosis of acute heart failure associated with nonocclusive mesenteric ischemia was retained. A review of the pertinent literature is presented.

## 1. Introduction

Heart failure (HF) patients commonly present with typical signs like fatigue and dyspnea along with lower legs edema when there is right ventricular involvement. Morbidity and mortality from HF are significantly higher when the diagnosis is delayed because of atypical presentation [1]. Nonocclusive mesenteric ischemia (NOMI) is a rare condition complicating the course of heart failure, and it is probably underdiagnosed and is associated with a high mortality rate [2]. Due to its dynamic and intermittent vasospastic physiopathology, NOMI is still a challenging diagnostic concern, and so far there is no standard diagnostic test of sufficient sensitivity and specificity [3]. Early diagnosis of NOMI is essential, also identification of triggering factors is critical to improve outcome [2, 3]. We present a case of severe heart failure presenting with nonspecific gastrointestinal (GI) symptoms compatible with NOMI.

## 2. Case Presentation

A 32-year-old male patient with no past medical history presented to the emergency room with abdominal pain, nausea, and vomiting. The patient had “normal” bowel movements and reported having these recurrent symptoms for the last 3 months. The patient was managed on an outpatient basis for nonspecific functional GI disorders and was treated as such with oral phloroglucinol and metoclopramide. 

**Figure 1:**
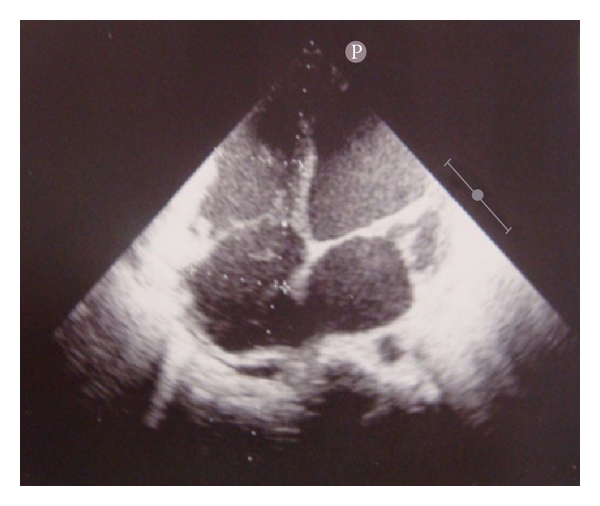
Cardiac echogram (apical view) showing severe dilation of 4 cavities.

Initial assessment in the emergency room revealed a blood pressure at 70/40 mm Hg, a heart rate at 120 bpm, cold extremities and general pallor, mild pulmonary rales, abdominal bloating, and no lower leg edema. There was no signs of acute surgical abdomen. Electrocardiogram showed sinus tachycardia with narrow QRS and diffuse nonspecific ST/T changes, chest X rays showed cardiomegaly, and emergent cardiac echogram showed severe hypokinetic dilated cardiomyopathy (Figure 1) with ejection fraction at 25%, normal pericardium, and no significant intrinsic valvular disease. 

The abdominal computed tomography (contrast-enhanced) scan showed nonspecific findings: no peritoneal effusion, no organic intraabdominal disease documented, and slow progression of contrast material attributed to the poor hemodynamic condition. Mesenteric angiography was not performed due to the unstable hemodynamic condition and the lack of consent.

 Laboratory findings revealed negative troponin, mild leucocytosis, serum sodium at 120 mmol/L, creatinine at 1.7 mg/dL, brain natriuretic peptide (BNP) at 3535 pg/mL, glutamic oxaloacetic transaminase (GOT) at 52 U/L, glutamic pyruvic transaminase (GPT) at 73 U/L, lactate dehydrogenase (LDH) at 271 U/L, and serum lactate at 4.8 mmol/L. Amylase, lipase, cortisol level, and other routine laboratory results were all within normal limits.

In the evolution, abdominal pain was resistant to conventional analgesics and antispasmodics, and it was only relieved when the hemodynamic condition improved. Also, during the intensive care unit stay, there were paroxysms of recurrent abdominal pain concomittant with episodic worsening of the hemodynamic condition. Of note, the patient needed intravenous positive inotropic agents for 14 days before the hemodynamic condition stabilized.

## 3. Discussion

This case shows an atypical presentation of HF consisting of abdominal syndrome compatible with NOMI. NOMI is a functional mesenteric circulatory disorder caused by a hypoperfusion state, it was first described by Ende [4] in patients with severe heart failure. Intestinal vasospasm related to decreased cardiac output and persistent low perfusion state is thought to be the underlying mechanism of NOMI [5, 6]. Early symptoms are often vague and in many cases, and the disease may progress to an advanced stage before a definitive diagnosis is established. The condition usually starts with nonspecific symptoms, consisting generally of mild abdominal pain that gradually progresses in severity, also bloating sensation, nausea, and vomiting may develop and evoke a subocclusion or occlusion [5]. 

The first issue to raise in this case report is that the presenting GI syndrome is atypical as a presentation of HF; this can be misleading and can explain the subsequent delay in the diagnosis and management of both HF and NOMI. Therefore, NOMI should be evoked every time that unexplained persistent GI symptoms manifest in the setting of a poor hemodynamic condition. 

 The second issue to raise is that definitive diagnosis of NOMI is often difficult, it is based essentially on clinical settings, and abdominal computed tomography with helical angiography is helpful when feasible but can be deceiving or unreliable because of the dynamic, functional, and spastic pattern of the disease [3, 5]; also angiography may not be achievable due to its complexity and invasiveness in the setting of unstable hemodynamic condition, and if performed, the spasm may be relieved, thereby missing the opportunity of accurate diagnosis [6, 7]. The activation of the renin-angiotensin system and vasopressin secretion are common in the setting of HF, and this phenomenon is probably involved in the physiopathology of NOMI, mainly the vasospasm of the splanchnic circulation; also the spasm may be triggered with the vasoactive and cardiotonic drugs given for HF [5]; among these, we list digitalis, furosemide, and ergotamine [3].

 Leucocytosis and elevated serum lactate are suggestive of NOMI in this setting [3], corresponding to the degree of intestinal ischemia and/or necrosis [8]; other laboratory findings are often non specific. 

 The absence of congestive HF symptoms like dyspnea and peripheral edema has been reported [9] in a series of patients despite severe depression of systolic function; the asymptomatic status was associated with a preserved autonomic function that was considered having a protective effect against the development of congestive HF symptoms despite increased levels of BNP. Given the physiopathological mechanism of NOMI that is related to a severe functional intestinal ischemia as a consequence of low perfusion state, NOMI is more imputed to forward HF rather than backward HF. 

 In the presented case, we hypothesized that NOMI is likely the cause of the abdominal syndrome according to the following criteria: patient presenting with severe HF, abdominal symptoms not relieved with classical analgesics but only with hemodynamic improvement, the syndrome relapsed when hemodynamic condition worsened, absence of specific organic intraabdominal pathology, mild increase in transaminases level. Other rare causes (i.e., porphyria, addison) of this abdominal presentation are to be evoked, but given the association between the relief of symptoms and improvement of the hemodynamic status, we refrained from performing extensive paraclinical tests in this setting. Mesenteric angiography is the standard test for the diagnosis of NOMI [3]; it shows in up to 90% of cases mesenteric vasoconstriction, impaired filling of intramural vessels, and regional vasospasm. 

## 4. Conclusion

NOMI is a critical condition that worsen the prognosis of patients with poor and unstable hemodynamic condition. Although it is a rare condition, it should be evoked in this clinical setting; also a multidisciplinary approach is of utmost importance in order to improve outcome.
